# Reflections on medical education in Brazil

**DOI:** 10.36416/1806-3756/e20250033

**Published:** 2025-03-18

**Authors:** Clystenes Soares Silva, José Roberto Jardim

**Affiliations:** 1. Disciplina de Pneumologia, Escola Paulista de Medicina, Universidade Federal de São Paulo - EPM-UNIFESP - São Paulo (SP) Brasil.

For most of the period during which Brazil was a colony of Portugal (1500-1815), physicians from Brazil were trained at universities in Europe. In 1808, the first medical school in Brazil was established in Rio de Janeiro by King Dom João VI (aka, King John VI of Portugal). That medical school is now part of the Federal University of Rio de Janeiro. After the Proclamation of the Republic, in 1889, when Brazil first became a democracy, several medical schools were created. In 1912, the Brazilian National Education Council was created and established standards for the implementation of medical education in the country. In 1930, the Brazilian government created the Ministry of Education and Public Health, from which curricular guidelines, supervision, and new courses were established.

An initial, fundamental question arises concerning public health in Brazil: does the country have an adequate number of physicians and medical schools? According to data from the Federal Council of Medicine, Brazil had approximately 575,000 physicians in 2024, equating to a ratio of 2.81 physicians per 1,000 population. This places Brazil ahead of countries such as China, Japan, and the United States. Over the past few decades, the number of physicians in Brazil has significantly increased (339%). During the same period, the population increased by 42%, from 144 million to 205 million. The rise in the number of physicians, which has outpaced population growth by a factor of eight, can be attributed to the nearly 400 medical schools in Brazil, second only to India, a country with a population that is six times larger. However, the high number of physicians does not translate to equitable distribution of the same. The number of physicians per 1,000 population in Brazil is only 1.73 in the northern region and 2.22 in the northeastern region, whereas it is 3.76 in the southeastern region. By 2028, Brazil is projected to have 3.63 physicians per 1,000 population, surpassing the density of 38 countries in the Organization for Economic Cooperation and Development. One noteworthy development is the anticipated increase in the annual number of medical graduates in the country, which could exceed 40,000 in the near future, driven by the growing number of medical schools. The persistent regional disparity in physician density has prompted discussions about its causes, which include low financial compensation, geographic remoteness, limited access to diagnostic tools, lack of opportunities for the children of physicians, and insufficient technical support. 

It is crucial to go beyond numerical analyses when assessing the medical workforce in Brazil, particularly given the high expectations for the performance of physicians and health care teams in the public system. This leads to another vital question: are Brazilian medical schools adequately preparing physicians to meet the needs of the public and private health care systems?

Medical education in Brazil is a six-year course. However, the rapid pace of scientific advancement has outstripped the traditional curricula. For instance, knowledge in nanotechnology doubles every 2-3 years and that in computer science doubles every 2 years. In medicine, the “half-life” of knowledge-defined as the time required for half of the information in a field to be replaced by new findings-is estimated at only 7-10 years because of advances in technology and biomedical research. These rapid changes highlight the need for medical education to be continually evolving.

The traditional lecture-based teaching model has gradually given way to problem-based learning, a student-centered pedagogical approach that fosters critical thinking, teamwork, and communication skills through problem-solving. However, some critics argue that problem-based learning may limit hands-on clinical experience, such as physical examinations and patient interactions. However, for both approaches, it has to be assumed that medical schools have adequate faculty, infrastructure, and opportunities for students to engage with patients in outpatient and hospital settings, alongside classroom learning of fundamental theoretical principles. However, the proliferation of new medical schools, particularly in smaller cities, raises concerns about their ability to recruit experienced faculty and provide high-quality education. In addition, disparities exist in teaching methodologies. Research-intensive institutions often focus on rare or complex cases seen at tertiary care centers, whereas medical schools in resource-limited settings and smaller cities may not expose students to sufficient complexity.

Currently, there is no standardized national licensing exam for medical graduates in Brazil, but the *Revalida* (“Revalidate”) exam, designed for foreign-trained physicians seeking to obtain a license to practice in Brazil, showed clearly concerning results in 2023: among 43 Latin-American universities that tested candidates, only 2 achieved a first-phase approval rate above 50%, whereas 18 had first-phase approval rates below 10%. 

Medical education in Brazil faces a number of challenges, including regional inequality; the rapid expansion of private courses; insufficient infrastructure for practical skills acquisition; and insufficient access to internship and residency programs. The reality of medical education in Brazil calls for the engagement of society, medical associations, and government bodies in the search for the best alternatives to minimize the problem, which is quite complex. Harmonizing factors such as the excessive number of medical schools, the quality of teaching, the competence of instructors, and the judicious use of new technologies, and thus training and qualifying physicians to practice responsibly in patient care, represent a challenge that cannot be dispensed with, otherwise the supreme duty of the physician will not be fulfilled, which is to provide proper care for the sick, thereby preserving sovereign respect for human dignity. There is no question about the importance of residency or an accredited internship for the necessary training of physicians. Unfortunately, even at large universities, there has been a significant decrease in the number of students who, upon graduating, complete their training to practice medicine. 

We cannot miss the opportunity to offer a medical course in which future professionals are prepared to stay up to date with scientific and technological advances, are familiar with new equipment used in diagnosis and surgical techniques, and are aware of advances in personalized therapy with medications that reach specific disease targets, such as biologics, and genomic techniques. Certainly, artificial intelligence is not a threat to physicians. On the contrary, artificial intelligence is an instrument capable of enhancing learning, allowing realistic simulation of clinical cases so that students can develop their technical skills and decision-making in a safe, controlled environment. It is also important to impart information about the management of financial resources, patient safety, organizational strategies, health policy planning, a broader vision of the problems, and the use of an efficient, accessible method of allocating available resources.

According to the Brazilian Institute of Geography and Statistics Synthesis of Social Indicators, 71.4% of physicians in Brazil have some connection with the Unified Health Care System, a large number of them in primary care. That raises two questions: Are six years of medical school enough to prepare students to work as physicians in a primary care setting?; and Is there an urgent need for medical residency to complement medical training? There may be several answers to and several pathways to answer those two questions. Starting with the second question, yes, medical residency is practically an obligatory complement for recent graduates, although there are not enough medical residency positions for recent graduates and this shortage of positions is likely to worsen as more physicians graduate in a short length of time. This brings us back to the first question, which is about the training of physicians and their ability to provide care in primary care or even in private practice. Knowledge in medical training should be such as to make physicians comfortable and confident in treating the most prevalent diseases in all specialties. In the short term, a small change in the medical education curriculum would achieve this goal. Students would be specifically exposed to the most prevalent diseases in each specialty, learning to recognize and treat them. In our specialty, pulmonology, graduating physicians should be very confident in recognizing and treating respiratory infections, asthma, COPD, smoking, pleural effusion, and lung cancer, being able to distinguish differential diagnoses for the most common respiratory symptoms. Other, difficult diagnoses, such as interstitial disease and pulmonary hypertension, would be referred to specialized centers. Increasing the number of positions in medical residency programs so that all new graduates can attend them will be possible only in the medium and long term.

Undoubtedly, there is a need to constantly update curricula and teaching methods to avoid a gap between dynamic knowledge and medical learning. However, and above all, interaction with human beings in order to transmit affection, welcome, and understanding, as well as to practice listening skills, is essential and can improve physician-patient relationships. Informing students on the subject of health management is quite relevant and absolutely necessary to prepare future physicians. With the aim of reaching those targets, medical education curricula should include competencies, skills, and knowledge that go beyond traditional clinical practice and are necessary to promote the sustainability of health care systems.
